# Identification of cuproptosis-associated subtypes and signature genes for diagnosis and risk prediction of Ulcerative colitis based on machine learning

**DOI:** 10.3389/fimmu.2023.1142215

**Published:** 2023-04-05

**Authors:** Dadong Tang, Baoping Pu, Shiru Liu, Hongyan Li

**Affiliations:** ^1^ Clinical Medical College, Chengdu University of Traditional Chinese Medicine, Chengdu, China; ^2^ Department of Anorectal Disease, Hospital of Chengdu University of Traditional Chinese Medicine, Chengdu, China

**Keywords:** ulcerative colitis, cuproptosis, machine learning, unsupervised hierarchical clustering, immune infiltration, nomogram

## Abstract

**Background:**

Ulcerative colitis (UC) is a chronic and debilitating inflammatory bowel disease that impairs quality of life. Cuproptosis, a recently discovered form of cell death, has been linked to many inflammatory diseases, including UC. This study aimed to examine the biological and clinical significance of cuproptosis-related genes in UC.

**Methods:**

Three gene expression profiles of UC were obtained from the Gene Expression Omnibus (GEO) database to form the combined dataset. Differential analysis was performed based on the combined dataset to identify differentially expressed genes, which were intersected with cuproptosis-related genes to obtain differentially expressed cuproptosis-related genes (DECRGs). Machine learning was conducted based on DECRGs to identify signature genes. The prediction model of UC was established using signature genes, and the molecular subtypes related to cuproptosis of UC were identified. Functional enrichment analysis and immune infiltration analysis were used to evaluate the biological characteristics and immune infiltration landscape of signature genes and molecular subtypes.

**Results:**

Seven signature genes (ABCB1, AQP1, BACE1, CA3, COX5A, DAPK2, and LDHD) were identified through the machine learning algorithms, and the nomogram built from these genes had excellent predictive performance. The 298 UC samples were divided into two subtypes through consensus cluster analysis. The results of the functional enrichment analysis and immune infiltration analysis revealed significant differences in gene expression patterns, biological functions, and enrichment pathways between the cuproptosis-related molecular subtypes of UC. The immune infiltration analysis also showed that the immune cell infiltration in cluster A was significantly higher than that of cluster B, and six of the characteristic genes (excluding BACE1) had higher expression levels in subtype B than in subtype A.

**Conclusions:**

This study identified several promising signature genes and developed a nomogram with strong predictive capabilities. The identification of distinct subtypes of UC enhances our current understanding of UC’s underlying pathogenesis and provides a foundation for personalized diagnosis and treatment in the future.

## Introduction

Ulcerative colitis (UC) is a chronic bowel disease with an unclear etiology and multi-factorial, multi-layered inflammation (1). The main symptoms of early UC include weight loss, frequent bowel movements, pain, and abdominal discomfort, and often more severe symptoms as the disease progresses. The incidence of UC has been increasing globally, but it remains difficult to cure, with only 15% of patients experiencing an aggressive course and some even developing dysplasia and colorectal cancer (2, 3). Although the exact cause of UC is unknown, it is believed to be linked to microbial imbalances, immune response, and genetic susceptibility (4, 5). Therefore, further exploring the exact etiology and pathogenesis of UC at the molecular level and accurately distinguishing UC subtypes can provide assistance for the diagnosis, treatment and prognosis of UC.

**Figure 1:**
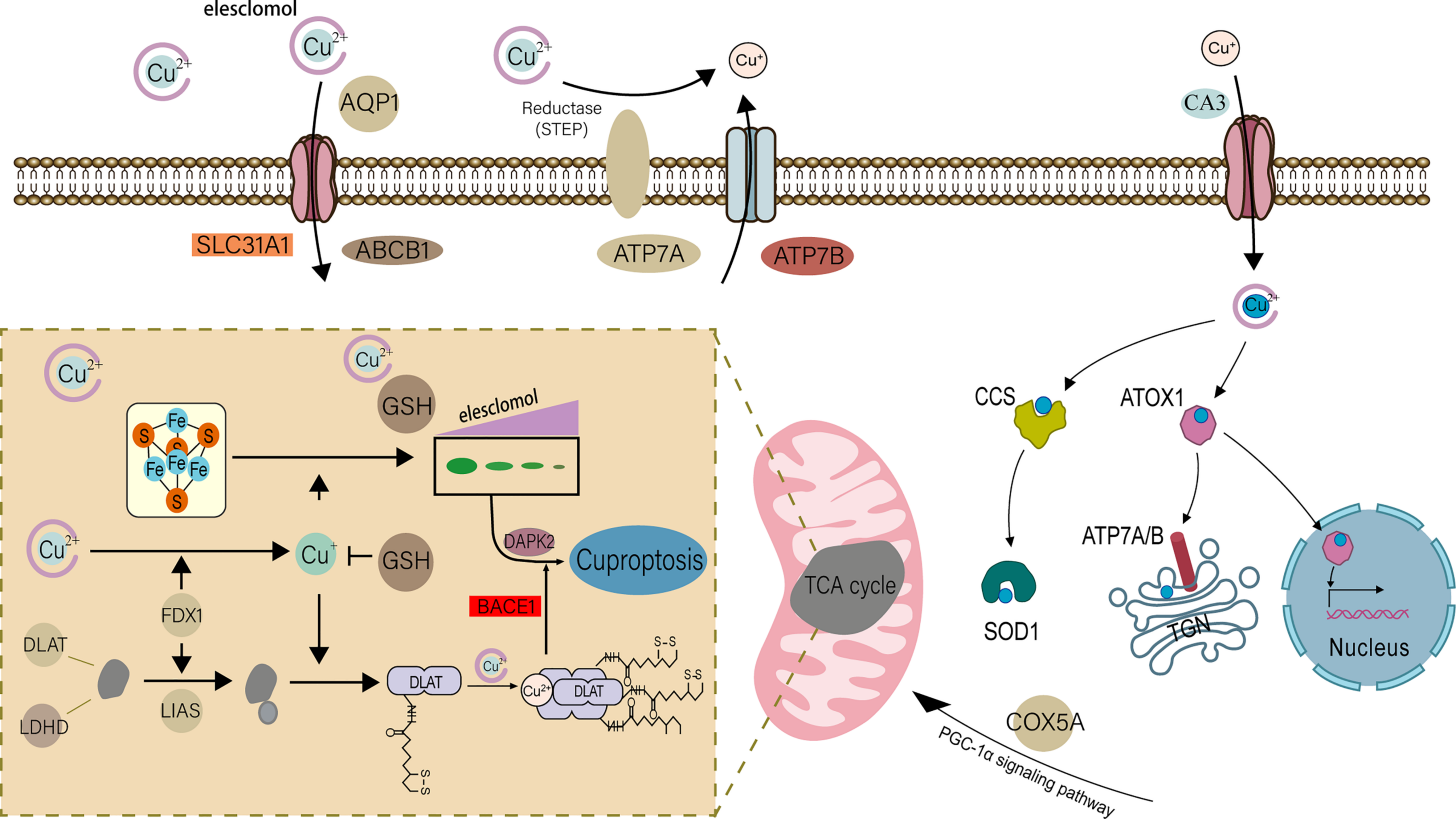
Schematic diagram of the mechanism of cuproptosis occurrence in cells.

For eukaryotes, copper participates in many important biological processes in the body and is an indispensable trace element (6). In a recent study, cuproptosis was discovered as a new form of controlled cell death, triggered by copper and different from cell death caused by oxidative stress, such as apoptosis and necroptosis (7). The specific mechanism is: the key protein in the tricarboxylic acid cycle in the mitochondrial respiration mechanism-fatty acylated protein directly combines with copper ions, resulting in the loss of iron-sulfur cluster protein, which in turn causes cell death (**Figure 1**) (7, 8). This study challenges the long-held belief that oxidative stress is a critical molecular mechanism of metal-induced toxicity and lends credence to the theory that mitochondria are complex regulators of cell death (9). In previous studies, in addition to normal programmed death, the occurrence of UC was found to be closely related to special cell death methods such as ferroptosis (10, 11). Previous studies have found that most UC patients have obvious copper metabolism abnormalities (12–14). Correspondingly, in some studies, copper is considered to be a key element in the body’s immune regulation mechanism (15–17). It has also been found that in the gut, mitochondrial metabolism and function play a key role in immune cell activation (18), and this is how copper ions direct cell death. Therefore, we believe that cuproptosis is closely related to the pathophysiology of UC.

In summary, in order to explore the pathogenesis of UC and the involvement of cuproptosis in the development of UC, we collected microarray datasets containing UC and normal tissues from the Gene Expression Omnibus (GEO) database (19). We then performed differential analysis and intersected with cuproptosis-related genes. We used three machine learning algorithms to screen out 7 signature genes, and then developed a novel nomogram based on the 7 signature genes to aid in the early detection of UC. In addition, we conducted an unsupervised cluster analysis of 298 UC patients based on 7 signature genes to reveal the heterogeneity among UC patients and determine a new UC classification. Finally, we explored the biological functions and immune mechanisms associated with each UC cluster using bioinformatics methods.

## Materials and methods

### Data collection and processing

We searched the GEO database using the keyword “ulcerative colitis” and vetted the dataset based on stringent inclusion/exclusion criteria. The inclusion criteria are: (a) only healthy control samples and UC patients’ genome-wide expression profiles are present in the datasets; (b) there are at least 10 UC samples and 10 healthy control samples in each dataset; (c) the test specimens in the datasets should be derived from human colon mucosal biopsy samples. The exclusion criteria are as follows: (a) the datasets contain samples of inflammatory bowel diseases other than UC. Finally, three microarray datasets were selected from GEO according to the inclusion/exclusion criteria. Three datasets [GSE38713 (20), GSE87473 (21), and GSE92415 (22)] containing UC and normal tissues were downloaded from the GEO database using the GEOquery package (https://www.bioconductor.org/packages/3.1/bioc/html/GEOquery.html) of R software (version 4.2.1) (23), and a total of 298 UC and 55 non-UC healthy participants were collected for the study (**Table 1**). The probe IDs of each gene were mapped to gene symbols. When multiple probe IDs were associated with a single gene symbol, the gene’s expression value was determined by taking the average expression of the corresponding probe IDs. For further analysis, we performed background calibration, normalization, and log2 transformation on all data. To avoid batch effects, a unified GEO dataset was created using the comBat function of the Bioconductor “sva” R package (https://www.bioconductor.org/packages/release/bioc/html/sva.html) (24). In addition, based on the same rigorous screening steps and preprocessing methods, we downloaded the GSE87466 dataset for validation (21). The details of all training and test sets are provided in **Table 1**. On the other hand, using “Cuproptosis” as the key word, the cuproptosis-related genes were retrieved from the Molecular Signature (MsigDB) database (http://www.gsea-msigdb.org/gsea/msigdb/) and summarized with the cuproptosis-related genes in previous studies (7, 15). After removing duplicates, we obtained a total of 61 cuproptosis-related genes.

**Table 1:** Basic information about the microarray data set for ulcerative colitis.

GEO Dataset	Platform	Samples	UC	Control
Training set:				
GSE87473	GPL13158	127 colon biopsy samples	106 active UC samples	21 controls
GSE38713	GPL570	43 colon biopsy samples	8 inactive UC samples 22 active UC samples	13 controls
GSE92415	GPL13158	183 colon biopsy samples	162 active UC samples	21 controls
Validation set:				
GSE87466	GPL13158	108 colon biopsy samples	87 active UC samples	21 controls

### Identification and functional enrichment of differentially expressed genes associated with UC

In the combined dataset, we screened out differentially expressed genes (DEGs) between UC and normal samples with |log2 fold change (FC)| > 0.3 and adjust p-value < 0.05 as the threshold, and visualized the results using the “limma” R package (https://www.bioconductor.org/packages/3.0/bioc/html/limma.html) to draw volcano maps and heat maps (25). Afterwards, we performed Gene Ontology (GO) enrichment analysis and Kyoto Encyclopedia of Genes and Genomes (KEGG) pathway analysis on the DEGs using the “clusterProfiler” package in R (https://www.bioconductor.org/packages/3.1/bioc/html/clusterProfiler.html) (26).

### Immune cell infiltration analysis

Due to the special immune pathogenesis of UC, understanding the infiltration of immune cells has irreplaceable significance for understanding disease progression and treatment. As an extension of the GSEA method, single-sample gene set enrichment analysis (ssGSEA) is widely used in bioinformatics studies related to immune infiltration. Therefore, we assessed the relative infiltrate abundance and immunological signature of all UC and normal tissue samples using the “GSVA” package (https://www.bioconductor.org/packages/3.1/bioc/html/GSVA.html) in R based on the ssGSEA algorithm (27, 28).

### Identification of signature genes by machine learning

We intersected cuproptosis-related genes with DEGs, and the overlapping genes were defined as differentially expressed cuproptosis-related genes (DECRGs). To identify potential biomarkers for UC, DECRGs were further screened using three machine learning algorithms: Least Absolute Shrinkage and Selection Operator (LASSO), Support Vector Machine Recursive Feature Elimination (SVM-RFE), and Random Forest (RF). Among them, the Least Absolute Shrinkage and Selection Operator (LASSO) algorithm finds the best model by introducing λ (lambda, also known as the penalty value, or shrinkage operator), at the same time, a penalty function is generated to compress the regression coefficients of the variables in the regression model, so eliminating the major covariance problem and preventing overfitting. The package “glmnet” (https://cran.r-project.org/web/packages/glmnet/) is used to implement this in R software (29). Support vector machine recursive feature elimination (SVM-RFE) based on the “caret” package (https://cran.r-project.org/web/packages/caret/) is an extremely powerful algorithm that assigns corresponding weights to two variables through the SVM algorithm (30, 31), and obtains smaller and smaller feature subsets through recursive screening, and use the RFE algorithm to select the optimal feature subset, and then obtain the optimal variables through 10-fold cross-validation. The Random Forest (RF) algorithm based on the “randomForest” package (https://cran.r-project.org/web/packages/randomForest/) (32)was designed to predict continuous variables and provide little fluctuating predictions for more accurate UC biomarkers. Subsequently, we observed the prediction accuracy of the feature genes obtained from the three machine learning algorithms in the validation set, and visualized the results as ROC curves, and then plotted the cumulative residual distribution of the three models to compare the screening performance of the three together.

### Construction and validation of the predictive model

The “gglot2” package (https://sourceforge.net/projects/ggplot2.mirror/) was used to build the nomogram of the signature gene-based UC risk prediction model (25), and the “pROC” R package (https://cran.r-project.org/web/packages/pROC/) was used to perform receiver operating characteristic (ROC) analysis to further evaluate the ability of the prediction model (33). Based on the “pacman” package (https://cran.r-project.org/web/packages/pacman/) (34), a calibration curve of the nomogram was drawn to verify its accuracy. Decision curve analysis (DCA) was performed using the “limma” package of R software to evaluate the clinical predictive value of the model. Finally, based on GSE87466 data set, calibration curve and ROC analysis were used to further evaluate the prediction performance of the prediction model.

### Identification of cuproptosis subtypes in ulcerative colitis

First, we classified 298 UC tissue samples into various clusters using an unsupervised hierarchical clustering analysis based on the 7 hallmark genes identified by machine learning (35). Principal component analysis (PCA), cumulative distribution function (CDF) curves, and consensus cluster scores were used to determine the optimal number of clusters. Subsequently, based on three different databases, we performed gene set variation analysis (GSVA) on the pathways of UC in different subclusters of cuproptosis discovered by cluster analysis (27), and drew a heat map to visualize the results. In addition, we evaluated the immune microenvironment of two different clusters to compare the differences in immune cell infiltration between them. Finally, we performed differential expression analysis of two gene subclusters of UC in cuproptosis, and functional enrichment analysis of differential genes to describe their biological functions. All the above analysis were performed using the corresponding packages (“ConsensusClusterPlus” (https://www.bioconductor.org/packages/2.7/bioc/html/ConsensusClusterPlus.html) (35), “limma”,”clusterProfiler”,”GSVA” and “GSEABase” (https://www.bioconductor.org/packages/3.0/bioc/html/GSEABase.html) (36) packages) in the R software. |log2 fold change (FC)| > 0.5 and adjust p value < 0.05 were considered statistically significant.

### Statistical analysis

All data processing, statistical analysis and plotting work were performed using R software (version 4.2.1). Differences between two groups were analyzed using Wilcoxon rank-sum test or Student’s t test. The Spearman correlation analysis was employed to determine correlations between immune cells and immune cells, as well as between immune cells and signature genes. The level of statistical significance was set at P value < 0.05.

## Results

### Data preprocessing and identification of DEGs

**Figure 2:**
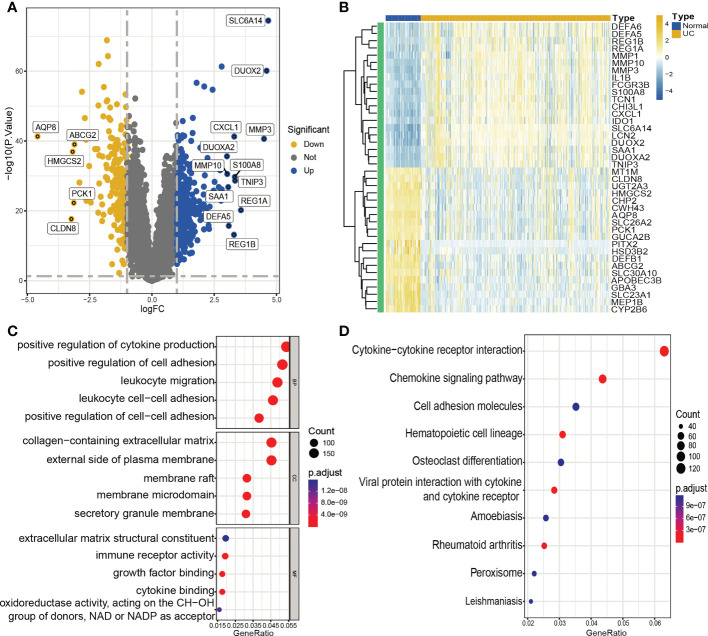
Identification of DEGs and functional annotation. **(A, B)**. Volcano plot **(A)** and heatmap **(B)** showing DEGs between UC and control groups. **(A)** Blue plot points represent upregulated DEGs, gray plot points represent genes with no significant difference, and yellow plot points show downregulated DEGs. **(B)** Each row of the heat map represents one DEG, and each column represents one sample, either UC or normal. **(C)** GO enrichment analyses of DEGs. BP, biological process; CC, cellular component; MF, molecular function. **(D)** KEGG enrichment analyses of DEGs.

The normalization, batch correction, and log2 transformation of the three microarray datasets produced a combined dataset, which was subjected to differential analysis. Filters of |log2 fold change (FC)| > 0.3 and adjusted p-value < 0.05 were employed during the analysis. The analysis resulted in the identification of a total of 4177 DEGs, including 2122 upregulated and 2055 downregulated genes. The visualization of the DEGs in the form of a volcano plot and heat map is presented in **Figures 2A, B**.

### Function and pathway enrichment analyses

We conducted GO enrichment analysis and KEGG pathway enrichment analysis to further investigate the possible biological roles of DEGs. The results of the analysis indicated that these DEGs were largely involved in several biological activities, including positive regulation of cytokine production, positive regulation of cell adhesion, leukocyte migration, and leukocyte cell-cell adhesion. Furthermore, the DEGs were enriched in pathways related to cytokine-cytokine receptor interaction, cell adhesion molecules, and the chemokine signaling pathway (**Figures 2C, D**).

### Immune cell infiltration analysis of UC

**Figure 3:**
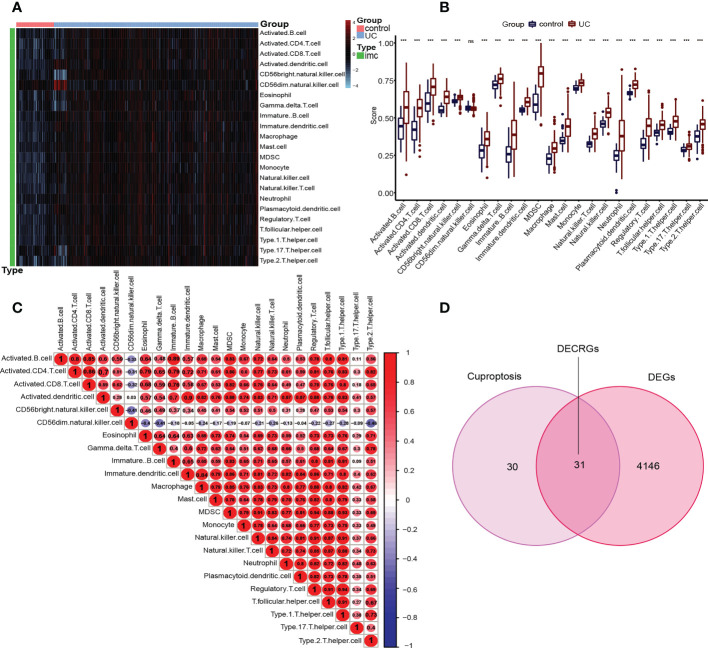
Immune infiltration in UC patients and controls and identification of DECRGs. **(A)** Each row of the heat map represents a type of immune cell, and each column represents one sample, either UC or normal. **(B)** Box plots depicting the infiltration levels of immune cells in UC and control groups. ***p < 0.001. ns, no significance. **(C)** Correlation heatmap depicting the correlations between distinct immune cell compositions. Red dots represent positive correlation, blue dots represent negative correlation, and the higher the absolute value of the number in the dot, the greater the correlation. **(D)** Venn diagram showing the DECRGs shared by DEGs and cuproptosis-related genes.

To deeply explore and reveal the immune mechanism during the onset and progression of UC, we used the ssGSEA method to score the immune cell infiltration between UC patients and controls to evaluate the immune cell infiltration landscape of UC. A heatmap of immune infiltration created based on ssGSEA scores is shown in **Figure 3A**. In the box plot (**Figure 3B**), we found that except CD56dim natural killer (NK) cells, the infiltration of the other 22 immune cells in UC tissues and the control group were significantly different (P<0.001). Interestingly, among the 22 types of immune cells, the immune scores of the UC group were all higher than those of the control group. The correlation analysis revealed a general correlation among different immune cells (**Figure 3C**). It is worth mentioning that, unlike other immune cells, CD56dim natural killer (NK) cell was negatively correlated with almost all other immune cells (except activated dendritic cell), which is a phenomenon worthy of attention.

### Identification of signature genes by machine learning

**Figure 4:**
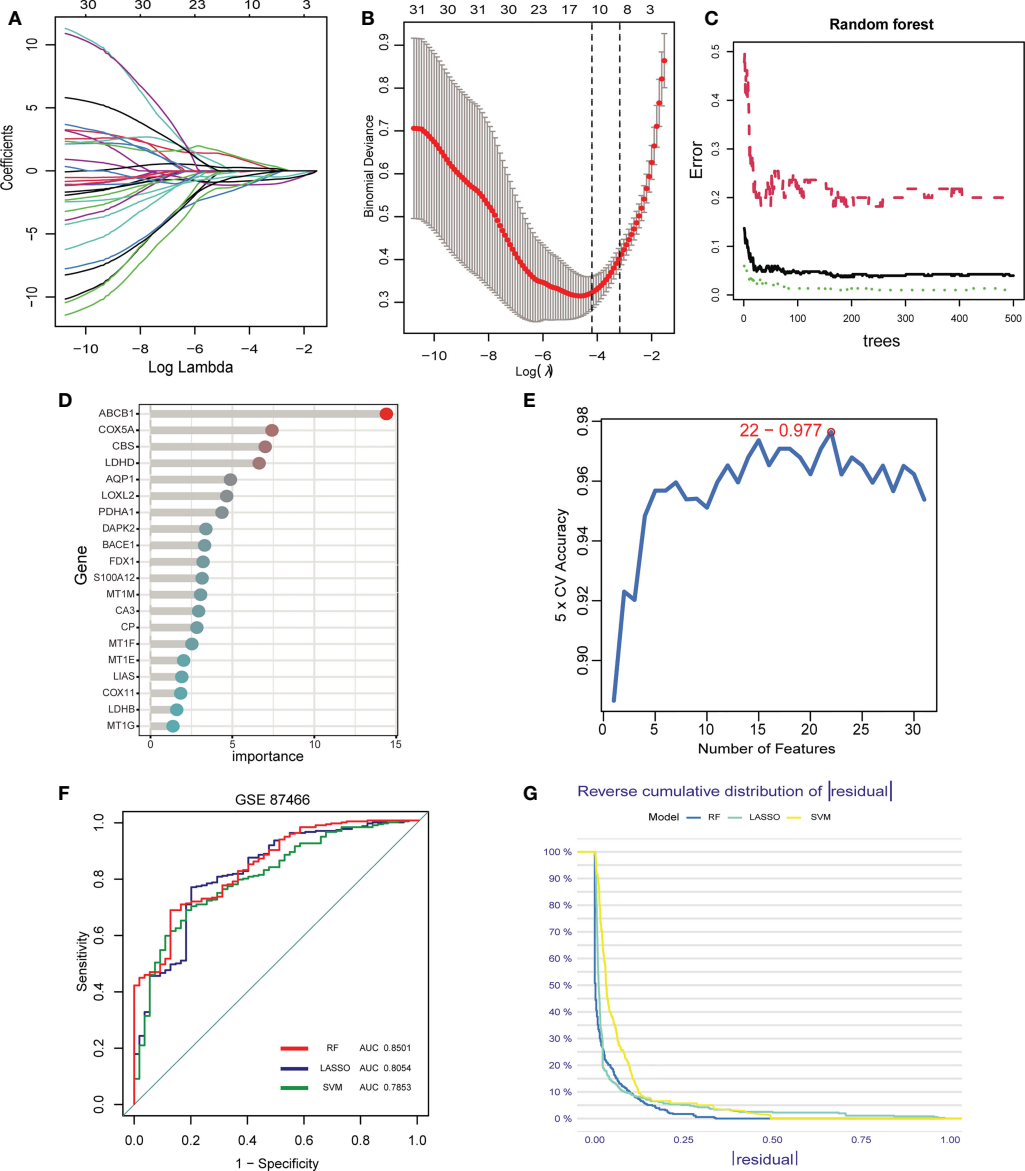
Screening of signature genes by machine learning. **(A-E)**. Construction of signature genes using LASSO regression **(A, B)**, SVM-RFE **(C, D)**, and RF algorithm **(E)**. **(F)** The ROC curves and AUC values demonstrate the screening performance of the three machine learning algorithms. **(G)** Cumulative residual distributions for the three machine learning algorithms (RF, LASSO, and SVM).

**Figure 5:**
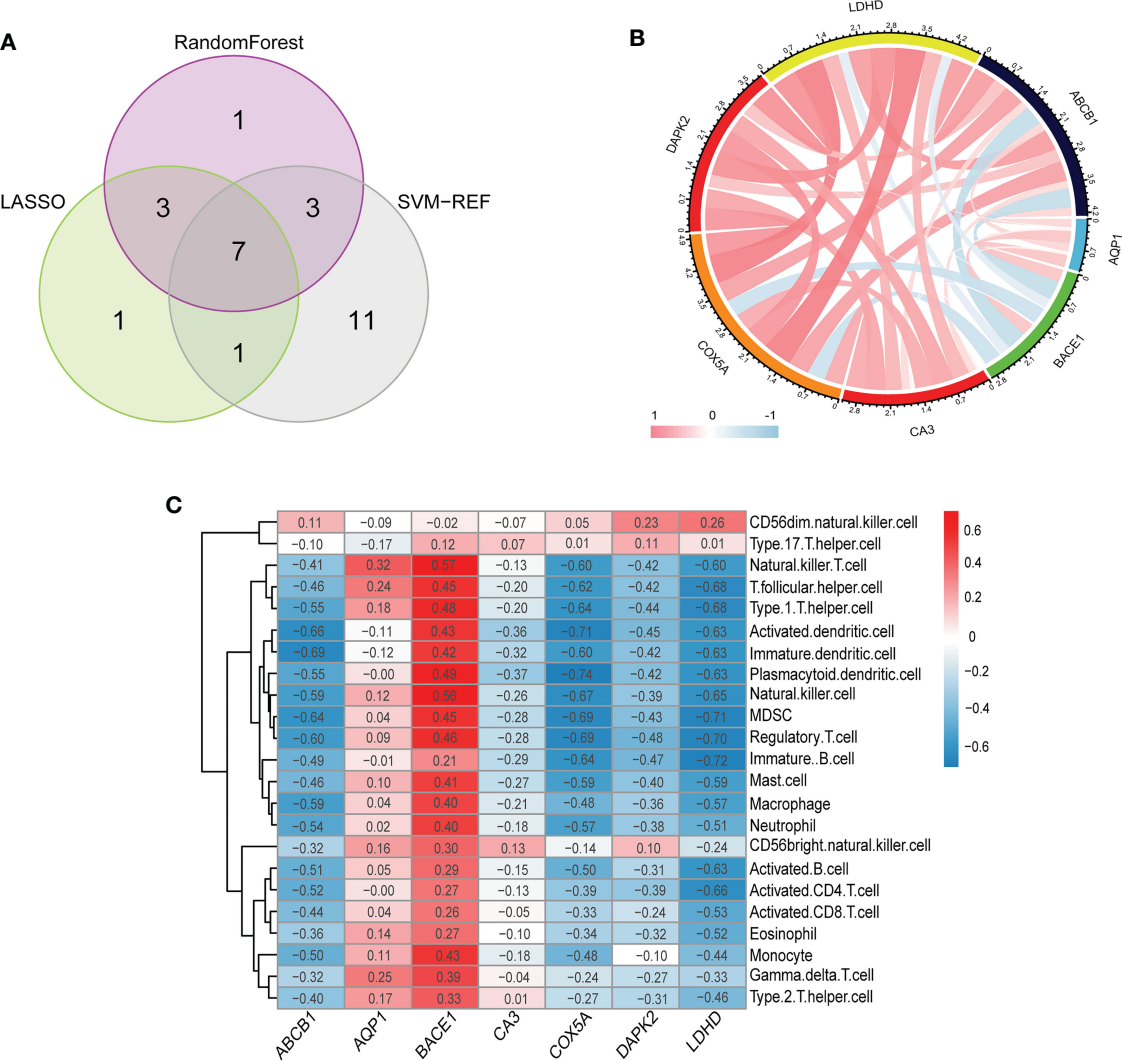
**(A)** The venn diagram shows the overlap of DECRGs between the three algorithms. **(B)** The chord diagram shows the relationship among the seven signature genes. **(C)** Correlation heatmap depicting the relationship between immune cell infiltration and signature genes. **(B, C)** Red indicates a positive correlation between two elements (signature gene and signature gene/signature gene and immune cells), blue indicates a negative correlation, the darker the color/the greater the absolute value of the value, the higher the correlation.

Inspired by previous research results, we used rigorous machine learning methods to screen the cuproptosis-related signature genes of UC. First, we obtained a total of 31 DECRGs by intersecting the cuproptosis-related genes with DEGs (**Figure 3D**). Subsequently, we screened the cuproptosis-related signature genes of UC through three machine learning algorithms. For the LASSO algorithm, we found the optimal lambda value to be 0.009 after ten-fold of cross-validation. Twelve potential signature genes with non-zero coefficients were subsequently screened out (**Figures 4A, B**). According to the results of the RF algorithm, we identified 14 genes with relative importance greater than 2.5 as signature genes (**Figures 4C, D**). For the SVM-RFE algorithm, the classifier reaches the minimum error when the number of features is 22, so we identify 22 valid signature genes (**Figure 4E**). Subsequently, we evaluated the performance of the three machine learning algorithms using the diagnostic ROC curves and AUC values of the signature genes screened by the three machine learning algorithms and the cumulative residual distribution curves. The results show that the feature genes screened by the three algorithms all have good prediction accuracy, which indicates that the performance of the RF algorithm is the best. The prediction accuracy of the feature genes screened by the RF algorithm is 0.8501, which is better than that of the LASSO algorithm (0.8054) and the SVM algorithm (0.7853) (**Figure 4F**). The vast majority of the residual inverse cumulative distribution curve of the RF model lies below the residual line of the LASSO and SVM models (**Figure 4G**), showing that the discrepancy between the predicted and true values of the RF is minimal and the performance is superior, while the LASSO algorithm has the second highest performance and the SVM algorithm has the relatively worst performance. Then, we identified 7 signature genes shared by the three machine learning algorithms by taking the intersection of the results, including LDHD, ABCB1, AQP1, BACE1, CA3, COX5A, and DAPK2 (**Figure 5A**). All seven signature genes have strong interactions, as shown in **Figure 5B**. In addition, the heat map of signature gene and immune cell correlations showed that all seven signature genes were correlated with immune cells to varying degrees. Notably, ABCB1, COX5A, DAPK2, and LDHD were strongly negatively correlated with most immune cells, whereas BACE1 was strongly positively correlated with most immune cells (**Figure 5C**).

### Establishment and validation of the signature gene-based nomogram for predicting UC

**Figure 6:**
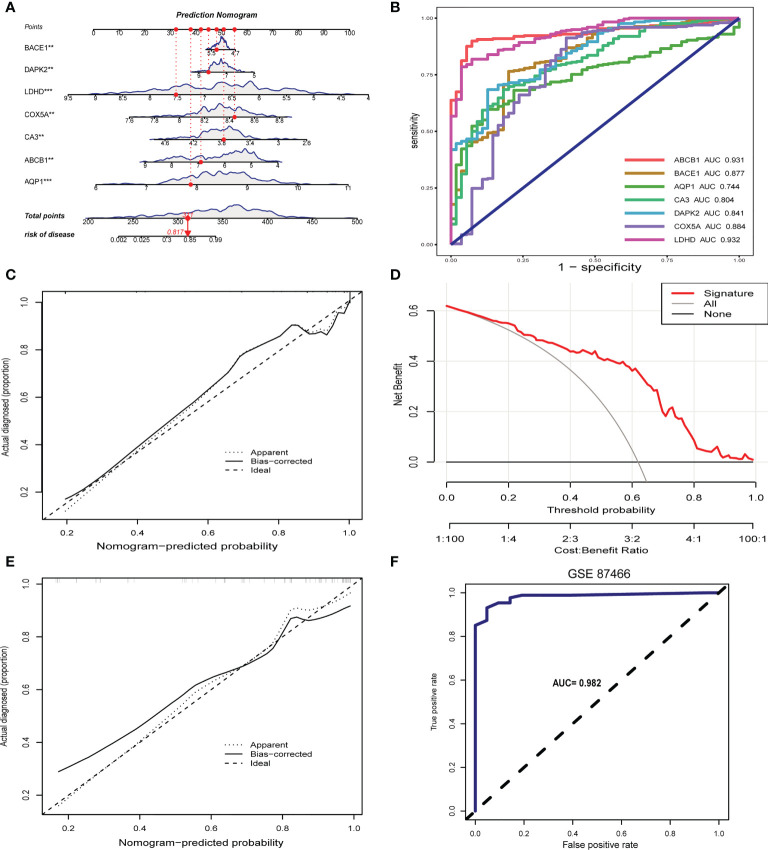
Construction and validation of the signature gene-based nomogram. **(A)** Nomogram for predicting UC risk based on signature genes. **(B)** ROC curve of signature genes in UC diagnosis. **(C)** Calibration curve for the nomogram. **(D)** Decision curve showing the clinical value of nomogram. **(E)** Calibration curve of nomogram for predicting UC in the validation set. **(F)** ROC curve for the validation set. **p < 0.01, ***p < 0.001.

Considering the lack of tools for clinical UC prevention and early diagnosis, we developed a nomogram for predicting UC risk based on the signature genes screened by machine learning (**Figure 6A**). In the nomogram, each signature gene corresponds to a score, and the scores of all signature genes are added to obtain a total score. Different total scores correspond to different UC risk. In **Figure 6B**, the ROC curve demonstrates the outstanding diagnostic value of each signature gene in predicting UC. The calibration curves and clinical decision curves also show that the nomogram have excellent accuracy and net clinical benefit (**Figures 6C, D**). In addition, the calibration curve (**Figures 6E**) and ROC curve (**Figures 6F**) of the prediction model in the validation set show that it has outstanding prediction performance.

### Construction of cuproptosis subtypes in ulcerative colitis

**Figure 7:**
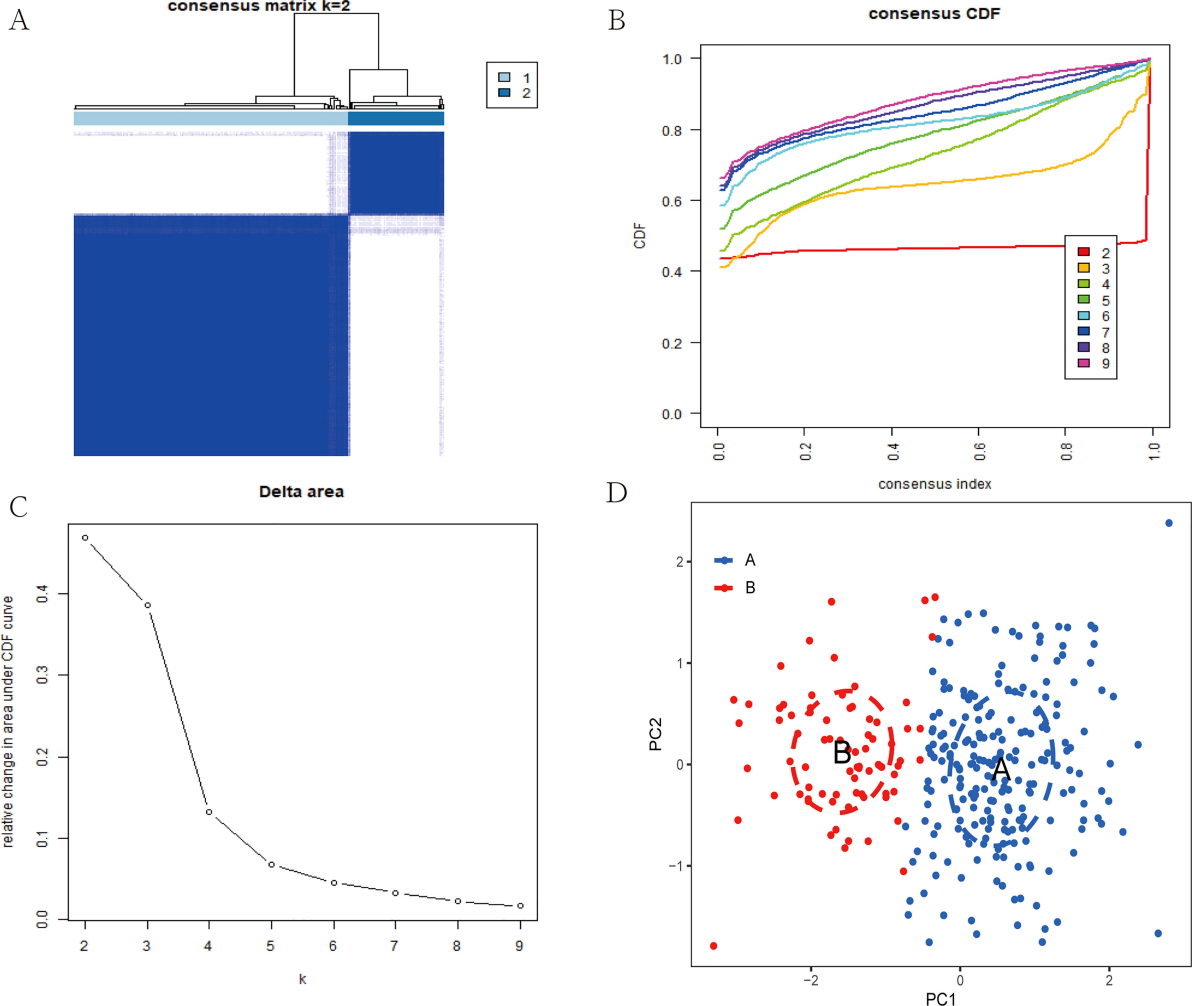
Construction of two cuproptosis subtypes in UC. **(A)** Consensus clustering matrix when k = 2. **(B)** Consensus CDF delta area curves when k = 2-9. **(C)** Relative alterations in the area under CDF curve. **(D)** PCA plot showing the distribution of the two subclusters.

**Figure 8:**
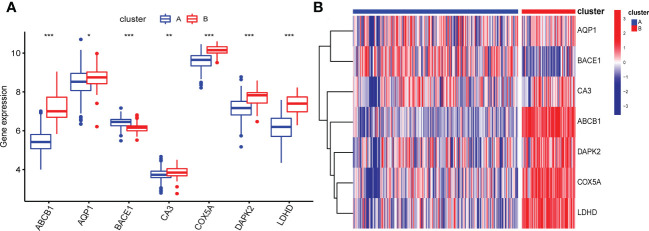
Box plot **(A)** and heatmap **(B)** showing differential expression of signature genes in the two cuproptosis subtypes. **(B)** Each row of the heatmap represents a signature gene, and each column represents a UC sample, either the A subtype or B subtype.*p < 0.05, **p < 0.01, ***p < 0.001.

Based on 7 signature genes, we clustered 298 UC samples by consensus clustering analysis. Finally, we determined that k=2 was the optimal grouping based on the consensus matrix graph, the CDF curve, and the relative changes in the area under the CDF curve (**Figures 7A-C**). Therefore, we finally obtained two cuproptosis subtypes of UC, named cluster A (n = 225) and cluster B (n = 73). Subsequently, we extracted the expression levels of all 61 cuproptosis-related genes in all samples of the two subtypes A and B (**Supplementary Tables 1**, **2**). In addition, principal component analysis (PCA) results further confirmed the clear distinction of the two clusters (**Figure 7D**). The boxplots and heatmaps of the two subtypes showed that the expression of seven signature genes had obvious heterogeneity in the two cuproptosis subtypes. Interestingly, the expression levels of ABCB1, AQP1, CA3, COX5A, DAPK2, and LDHD were significantly higher in cluster B than in cluster A, while the opposite was true for BACE1 (**Figures 8A, B**).

### Pathways activity and immune infiltration landscape between cuproptosis clusters

**Figure 9:**
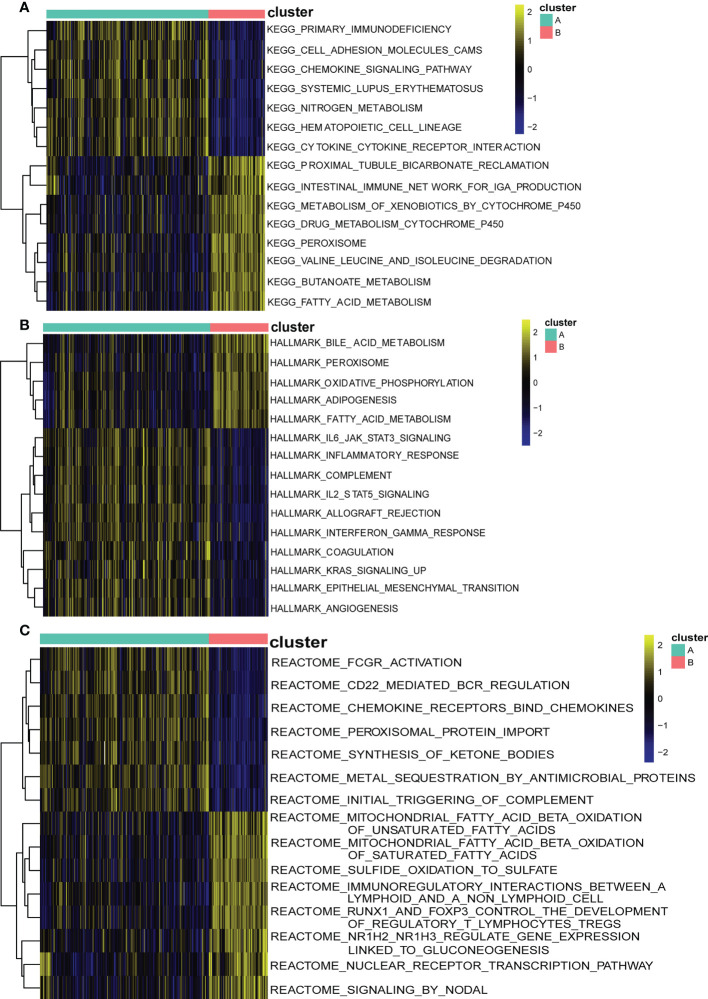
Biological characteristics of two distinct cuproptosis subtypes of UC revealed by GSVA. **(A)** Enriched pathways based on the KEGG pathway.**(B)** Enriched pathways based on the Hallmark pathway. **(C)** Enriched pathways based on the Reatcome pathway. Each row of the heatmap represents an enriched pathway, and each column represents a UC sample, either the A subtype or B subtype.

**Figure 10:**
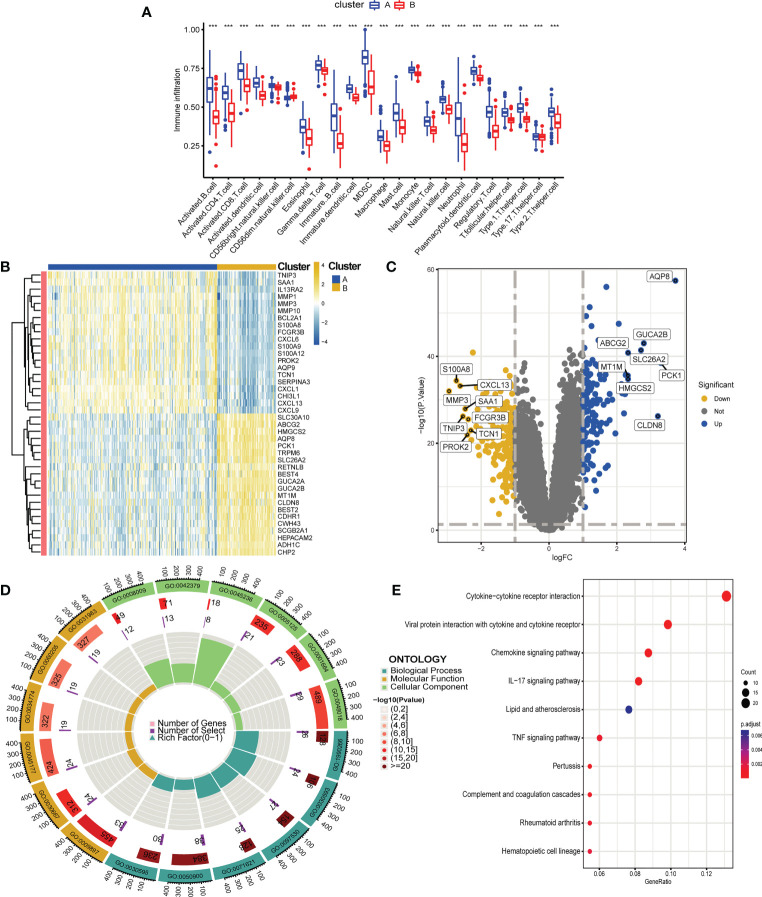
Differential analysis, functional enrichment analysis and immune infiltration analysis between two cuproptosis subtypes of UC, functional enrichment analysis and immune infiltration analysis. **(A)** Boxplot showing differences in immune infiltration between the two cuproptosis subtypes. **(B, C)**. Heatmap **(B)** and volcano plot **(C)** depicting DEGs between the two cuproptosis subtypes. **(B)** Each row of the heat map represents one DEG, and each column represents a UC sample, either the A subtype or B subtype. **(C)** Blue plot points represent upregulated DEGs, gray plot points represent genes with no significant difference, and yellow plot points show downregulated DEGs. **(D, E)**. GO **(D)** and KEGG **(E)** enrichment analyses of DEGs between the two cuproptosis subtypes. ***p < 0.001.

To explore the possible pathological mechanisms of each UC subtype and assess the difference in pathway activity between the two clusters, we further performed GSVA between the UC of the two cuproptosis subtypes. The results are shown in the heatmap, in cluster A, the expression of KEGG pathways associated with bicarbonate reclamation, peroxisome, butanoate and fatty acid metabolism was significantly decreased, while the expression of pathways associated with nitrogen metabolism, immunodeficiency and chemokine signaling were significantly elevated (**Figure 9A**). In addition, cluster B had higher Hallmark activity for bile acid metabolism, oxidative phosphorylation, and adipogenesis, and lower IL6-JAK-STAT3 signaling, KRAS signaling, and inflammatory response compared to cluster A (**Figure 9B**). While based on the reatcome pathway, the results of GSVA showed that the immunomodulatory interaction between lymphocytes and non-lymphocytes, nodal signaling and nuclear receptor transcription pathways and other related pathways were significantly enriched in cluster B. In contrast, Cd22 mediated BCR regulation, ketone body synthesis, and complement initial trigger enrichment in cluster A (**Figure 9C**). As shown in **Figure 10A**, the level of infiltration of 22 immune cells (except CD 56 dim NK cell) was significantly higher in patients in group A than in patients in group B, which coincided with the results of GSVA and the results of the immune infiltration analysis of DEGs. These results suggest that there are significant differences in the pathway activity, biological function and immune infiltration among different subtypes of UC. Therefore, different subtypes should be treated with different strategies. In addition, CD 56 dim NK cells showed their special infiltration in this study, so its role in UC deserves further investigation.

### Biological functional distinctions between cuproptosis clusters

We performed differential expression analysis to further investigate the differences in gene expression and biological activities between the two subclusters of cuproptosis. A total of 333 differentially expressed genes were identified using |log2 fold change (FC)| > 1 and adj. p < 0.05 as screening conditions. Among them, 198 were up-regulated genes and 135 were down-regulated genes, and we visualized the results using heat map and volcano map (**Figures 10B, C**). We then performed functional enrichment analysis of 333 differential genes, and the results of GO and KEGG enrichment analysis showed that these differentially expressed genes were mainly enriched in biological processes such as leukocyte chemotaxis, leukocyte migration, granulocyte chemotaxis, neutrophil chemotaxis and neutrophil migration, and related pathways such as chemokine signaling pathway, IL-17 signaling pathway and TNF signaling pathway, which is also consistent with the previous results of GSVA (**Figures 10D, E**).

## Discussion

UC is a chronic inflammatory bowel disease mainly manifested by mucosal inflammation and ulceration, and its most common site of disease is the colon (37). It is often referred to as a non-fatal cancer because of its severe long-term local and systemic symptoms and frequent recurrence. Currently, the diagnosis and treatment of UC is still limited by the available technology. The diagnosis of UC relies heavily on gastrointestinal endoscopy and mucosal histopathological biopsy, which will delay the timing of treatment for some patients with UC with atypical endoscopic signs or pathological features (38, 39). Therefore, the development of new diagnostic tools for the risk stratification of UC is of great significance. In addition, the current treatment efficiency of UC is also very low, and the complete cure rate is close to zero. For patients with mild to moderate UC without complications, surgical treatment is generally not given clinically, and only glucocorticoids are given to help relieve symptoms (40–42). This has indirectly led to the fact that most UC patients need to take medicine for life, which brings a heavy economic burden to their families (43, 44). Therefore, it is imperative to explore new therapeutic targets to improve and individualize treatment approaches.

In this study, we linked cuproptosis to UC, explored its possible pathogenesis, and created new possibilities for the diagnosis, treatment and prognosis of UC. First, we obtained the gene expression matrices of 298 UC patients and 55 healthy controls from three datasets in the GEO database, and finally identified 4177 DEGs. Functional enrichment analysis based on DEGs revealed that biological processes such as leukocyte adhesion and leukocyte migration were significantly enriched. Indeed, in previous studies (45), rapid recruitment and inappropriate retention of leukocytes has been shown to be a hallmark of all chronic inflammatory diseases, including UC, which is consistent with our study. As a result, we speculate that DEGs play an important role in UC through modulating leukocyte migration and adhesion. In addition, KEGG enrichment results showed that DEGs were mainly related to chemokine signaling pathways. All inflammatory responses inevitably contain one of the most prominent features: leukocyte infiltration. The recruitment and maintenance of leukocytes during inflammation requires complex signaling, of which the chemokine pathway is the most important. However, several previous studies (46–48) have shown that different cells can lead to different outcomes through the same signaling pathway. Therefore, we further hypothesized that leukocyte adhesion and migration through chemokine signaling pathway is a possible pathogenesis of UC.

In addition, the critical role of the immune response in the pathogenesis of UC has long been the focus of attention. We therefore assessed differences in immune infiltration between UC and normal tissues using ssGSEA (28). The results showed that the expression levels of almost all immune cells in UC were significantly higher than those in the normal group, which was the same as the results of many previous studies (49, 50). It is worth mentioning that our study found that CD56 dim NK cells were the only cells that did not differ between the two. As a fully mature NK cell, CD 56 dim NK cells account for 90% of peripheral blood NK cells and mainly play a role in mediating cytotoxicity (51, 52). In previous studies (53), CD 56 dim NK cells have been found to be associated with a number of autoimmune diseases, including ankylosing spondylitis, systemic lupus erythematosus, Behçet’s disease, multiple sclerosis, and type I diabetes. In addition, a 2018 study showed that phenotypic changes in circulating immune cell subsets such as CD 56 dim NK cells are an important cause of the occurrence and progression of colorectal cancer (54). Overall, although our study suggests that CD 56 dim NK cells are associated with the occurrence and development of UC, the specific mechanism needs further study. In previous studies, the differentially expressed genes in UC were mainly concentrated in the immune process, and our ssGSEA and enrichment analysis results provided new evidence for this conclusion.

Cuproptosis, a newly discovered form of cell death (7, 8), still needs further investigation for its potential association with UC given that UC patients have been found to have copper accumulation in previous studies (14, 55). Therefore, we intersected DEGs and cuproptosis genes, and then used machine learning methods for further screening, and finally obtained 7 signature genes related to cuproptosis (ABCB1, AQP1, BACE1, CA3, COX5A, DAPK2, and LDHD). The ABCB1 gene encodes for the P-glycoprotein (P-gp) protein, which is a member of the ATP-binding cassette (ABC) transporter family. P-gp is a multi-drug resistance protein that is involved in the efflux of a wide range of compounds from cells, including xenobiotics and drugs (56). Recent studies have suggested that P-gp can play a role in cuproptosis by regulating copper efflux from cells. Particularly, P-gp has been shown to control copper homeostasis in the cell and stop oxidative stress and cell damage caused by copper (57). In a previous study from Denmark (58), ABCB1 was found to be a “risk gene” for the development of inflammatory bowel disease. According to Anderson et al. (59), low ABCB1 gene expression may contribute to the development of intestinal disease by increasing intracellular exposure to oncogenic or inflammatory ABCB1 substrates. In addition, Cao et al. (60) found that the ABCB1 C1236T polymorphism was strongly associated with UC. Aquaporin 1 (AQP1) is a water channel protein that is expressed in various tissues, including the brain and lungs (61–63), where it plays a role in regulating water transport. AQP1 has been shown to be involved in the transport of copper ions into cells, and its expression has been found to be regulated by copper ions (64). In some cell types, AQP1 has been shown to be required for cuproptosis to occur. For example, in astrocytes, AQP1 has been shown to play a role in the transport of copper ions into the cell, leading to oxidative stress and cell death (65). In addition, Ricanek et al. (66) found that reduced AQP1 expression levels were the main cause of reduced water and glycerol absorption in the small intestine, which may further contribute to the development of symptoms such as diarrhea and malnutrition in UC. Carbonic anhydrase (CA) is a family of enzymes that catalyzes the reversible hydration of carbon dioxide to bicarbonate and protons. Studies have shown that CA enzymes play a crucial role in the regulation of cuproptosis and have been shown to regulate cuproptosis by modulating copper ion homeostasis within the cell (67, 68). Inhibition of CA enzymes has been shown to enhance cuproptosis, while overexpression of CA enzymes has been shown to protect cells from cuproptosis.Carbonic anhydrase 3 (CA3) is a zinc-containing metalloenzyme that catalyzes the reversible hydration of carbon dioxide and regulates cellular ion transport and pH homeostasis. Unlike other carbonic anhydrase family members, CA3 has 2 reactive sulfhydryl groups that are reversibly bound to glutathione *via* disulfide bonds and can protect cells from oxidative stress (69). According to Okada et al, CA3 in the colonic mucosa significantly inhibits the secretion of inflammatory cytokines and has a protective effect against the exacerbation of UC (70). In a previous study, BACE1 was pointed out to mediate the progression of Crohn’s disease by regulating cuproptosis (49). Moreover, previous studies have found that BACE1 inhibition can increase susceptibility to oxidative stress by promoting mitochondrial damage, but the exact mechanism is unclear (71). This has some similarity with the biological function of COX5A, which has been reported in previous studies to be involved in the process of aluminum-induced oxidative stress (72, 73), promoting reduced mitochondrial biogenesis by regulating the PGC-1α signaling pathway, which coincides with the biological process of cuproptosis-regulated cell death. DAPK2 is a member of the death-associated protein kinase (DAPK) family, and several studies have investigated the potential relationship between DAPK2 and copper apoptosis. For example, a study published in 2019 suggested that DAPK2 may regulate copper apoptosis by regulating cellular redox homeostasis and regulating the accumulation of copper ions in cells (74). In addition, its involvement in UC development through classical pathways such as Hippo and Ephrin receptor signaling pathways has been identified in many previous studies (75, 76). The LDHD gene, also known as L-Lactate Dehydrogenase D, is involved in the metabolic pathway that converts pyruvate to lactate. It has been shown that LDHD is involved in the regulation of copper apoptosis by controlling intracellular copper levels through involvement in the fatty acylation process of proteins in the TCA cycle (77, 78). LDHD is expressed in intestinal epithelial cells and is involved in the regulation of oxidative stress and cellular metabolism, and it is believed that altered LDHD gene expression may lead to oxidative stress and altered cellular metabolism, resulting in increased intestinal inflammation (79), but the exact mechanism of its association with the development of UC is not fully understood.

Subsequently, we conducted a systematic bioinformatics analysis on these 7 signature genes to explore the inner relationship between gene-gene and gene-disease. First, we found a general correlation (positive or negative correlation) among the signature genes, suggesting that synergistic or antagonistic interactions between them may be the underlying cause of the occurrence and development of UC. Subsequent studies on the association between characteristic genes and immune cells showed that BACE1 was positively correlated with 22 immune cells (except CD 56 dim NK cells), while ABCB1 was just the opposite, and it was negatively correlated with 22 immune cells (except CD 56 dim NK cells), suggesting that abnormal infiltration of CD 56 dim NK cells may play an important role in the progression of UC. In addition, COX5A, DAPK2 and LDHD were all strongly negatively correlated with the majority of immune cells. Clinically, the diagnosis of the vast majority of UC patients is usually after the occurrence of intestinal mucosal injury. The establishment of a prediction model tool at the gene level and the detailed classification of UC can not only improve the accuracy of clinical diagnosis and treatment of UC, but also provide strategies for preventing the occurrence of the disease in the future. Therefore, we established a predictive model of UC based on the above seven signature genes, which has been verified to have good predictive ability and clinical benefits. Subsequently, we identified two cuproptosis-associated clusters of UC based on signature genes using unsupervised cluster analysis. In cluster A, the expression of signature genes was significantly lower than that in cluster B. Additionally, cluster A exhibited higher expression of immune cell infiltration compared with cluster B. Cluster B had higher amounts of immune-related pathways and lower levels of metabolic-related pathways in the functional analysis of clusters, while cluster A had the opposite. We identified differential genes between the two subtypes of cuproptosis in UC, and finally screened out 333 differential genes. In the subsequent functional enrichment analysis, we found that the differential genes between the two subtypes were mainly enriched in inflammation-related biological processes and classic inflammation-related pathways such as chemokine signaling pathways, IL-17 signaling pathways, and TNF signaling pathways. These results are consistent with previous research conclusions (47, 80, 81). In conclusion, the systematic study on the cuproptosis subtypes and diagnostic markers of UC will help us better understand the pathogenesis of UC and provide a theoretical basis for its personalized diagnosis and treatment.

In this study, we screened out highly valuable signature genes through bioinformatics methods, and created a predictive model with accurate diagnostic capabilities. In addition, we also classified UC based on cuproptosis-related genes for the first time. These results may bring new perspectives for the early prevention and personalized diagnosis and treatment of UC. Nonetheless, several limitations need to be pointed out. First of all, since the data used in the above analysis are all from the same database, and there is a lack of original sequencing data, there may be unavoidable selection bias. Secondly, because the sample size is relatively small, and the potential influence of patients’ complications, gender, and age is ignored, a large clinical cohort prospective study is still needed to further study the potential of signature genes in predicting UC. In addition, the mechanism of action of these signature genes will be further elucidated through *in vitro* and *in vivo* experiments.

## Conclusion

In conclusion, we finally screened seven signature genes (ABCB1, AQP1, BACE1, CA3, COX5A, DAPK2, and LDHD) associated with UC and cuproptosis through a series of bioinformatics analyses, and a nomogram composed of these genes can effectively predict the risk of UC occurrence. In addition, for the first time, we have divided UC into two distinct subtypes, which improves understanding of the disease and can guide individualized treatment. Therefore, our study may provide new insights into exploring the heterogeneity of clinical manifestations and prognosis of UC, and provide a theoretical basis for risk stratification and individualized diagnosis and treatment of UC in the future.

## Data availability statement

The datasets presented in this study can be found in online repositories. The names of the repository/repositories and accession number(s) can be found within the article/**Supplementary Materials**.

## Author contributions

DT designed the study and drafted the initial manuscript. BP and HL collected the original data and finished the analysis. HL refined the drawing of the pictures and provided constructive comments on the writing of the manuscript. SL directed the research and proposed changes to the manuscript. All authors contributed to the article and approved the submitted version.

## Supplementary material

Supplementary Table 1 Expression levels of cuproptosis -associated genes in samples from subtype A of Ulcerative colitis.

Supplementary Table 2 Expression levels of cuproptosis -associated genes in samples from subtype B of Ulcerative colitis.
